# Supplementary material for: Measuring sub-nanometer undulations at microsecond temporal resolution with metal- and graphene-induced energy transfer spectroscopy
Source: Nat Commun. 2024 Feb 27;15:1789. doi: 10.1038/s41467-024-45822-x (PMC10899616; doi:10.1038/s41467-024-45822-x)
Supplement: Supplementary file 1 — Supplementary Information [file 41467_2024_45822_MOESM1_ESM.pdf]

Supplementary Information

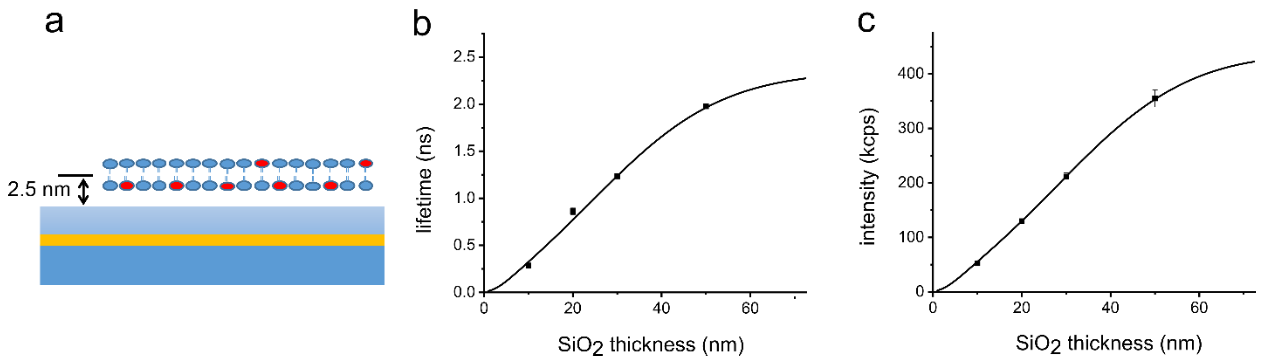

Supplementary Figure 1: Experimental verification of theoretical calibration curves. (a) Schematic of a MIET calibration experiment used to validate the theoretical calculations: a glass cover slide is covered with a 20 nm thick gold layer that is covered with SiO_2 spacer layer of varying thickness. On top of that, a supported lipid bilayer (SLB) with fluorescently labeled lipids is prepared. In panels (b) and (c), the solid curves represent calculated MIET lifetime and brightness curves, respectively, for an electric dipole (DPPE-atto655) with an emission wavelength of 680 nm, a quantum yield of 0.36, a free space lifetime of 2.6 ns.[1] The refractive index of the dipole-embedding medium (aqueous buffer solution in experiments) was set to 1.33, and that of the SiO_2 spacer to 1.46. The experimental data in panels (b) and (c) were measured with SLBs comprising DOPC and 0.001% DPPE-Atto655 on four substrates with different silica spacer thicknesses (10 nm, 20 nm, 30 nm, and 50 nm). The error bars represent the standard deviations of the measurements. Source data for (b) and (c) are provided as a Source Data file.

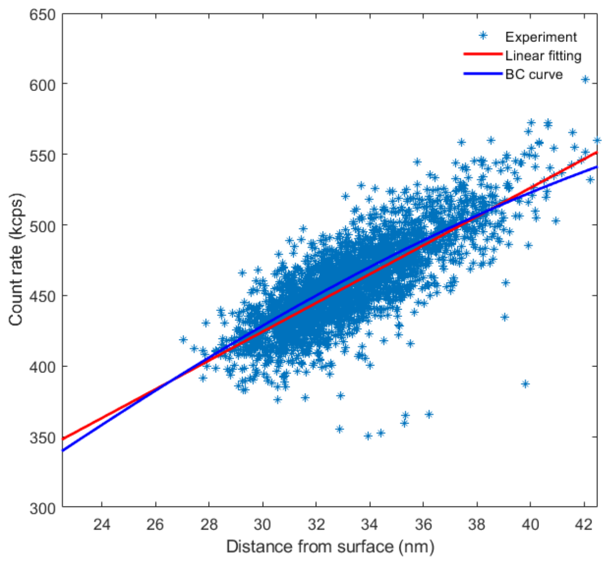

Supplementary Figure 2: Check of linearity for the relation between height and fluorescence intensity. Scatter plot showing the correlation between measured intensities and respective height values as calculated from fluorescence lifetimes, as obtained for a deflated GUV. The experiment lasted for 300 s and was divided into 3000 bins of 100 ms time width. The red line represents a linear fit of the scatter plot data, and it has a slope of 10.2 kcps/nm. The blue line represents the theoretical brightness calibration curve, having a slope at a distance of 33 nm of 9.8 kcps/nm. Source data are provided as a Source Data file.

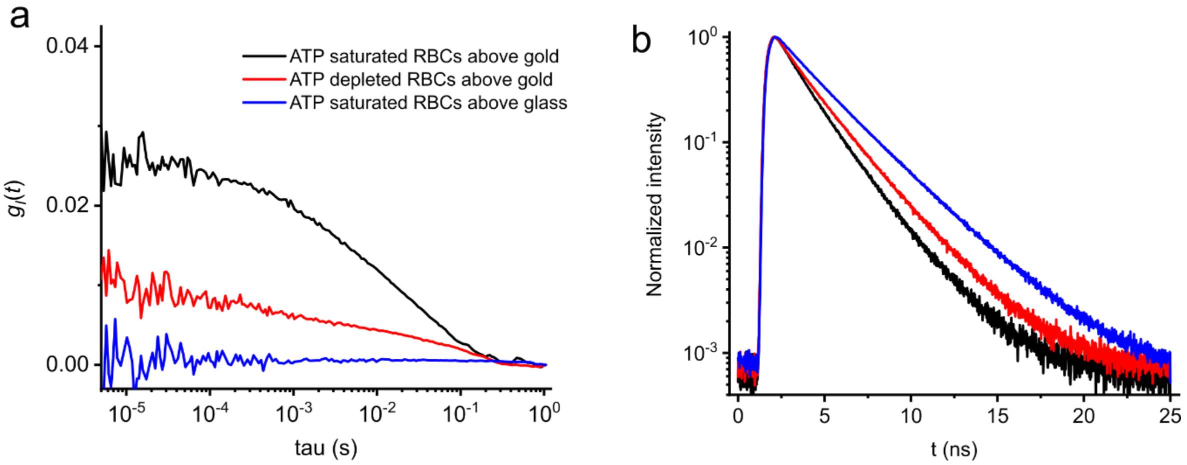

Supplementary Figure 3: Intensity correlations and lifetime decay curves for RBCs. (a) Intensity autocorrelation function, $g_I(t)$, measured at the rim of RBCs on different substrates and for ATP-saturated and ATP-depleted buffers. (b) Corresponding normalized fluorescence lifetime curves. The intensity correlation measurement at the glass surface showed almost no amplitude, indicating that the dye concentration used was sufficiently large to suppress any diffusion-related contribution to the correlation curve. Source data are provided as a Source Data file.

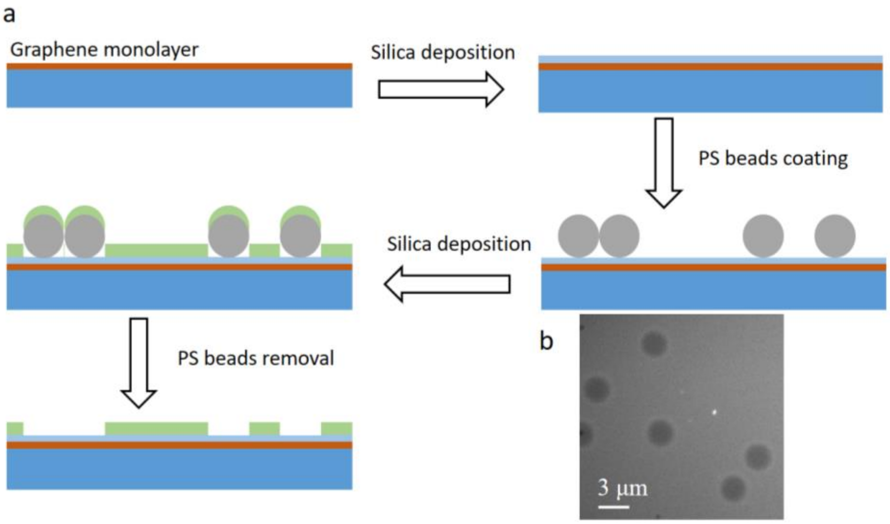

Supplementary Figure 4: Fabrication of graphene-pore substrate. (a) Schematic of GIET Substrate Fabrication for PSM Measurements: Initially, the graphene-coated coverslip is deposited with a 2 nm SiO_2 layer to safeguard the graphene monolayer. Following this, a 10 µL droplet of diluted (100 µg/mL) aqueous colloidal suspension is placed onto the surface and allowed to dry at room temperature. The PS-coated graphene/SiO_2 coverslip is further layered with the desired thickness of SiO_2. Finally, the polystyrene (PS) beads are eliminated using an acetone solution for 15 minutes. (b) SEM images of the pore-covered GIET substrate. The SEM experiment in (b) was repeated two times with similar results.

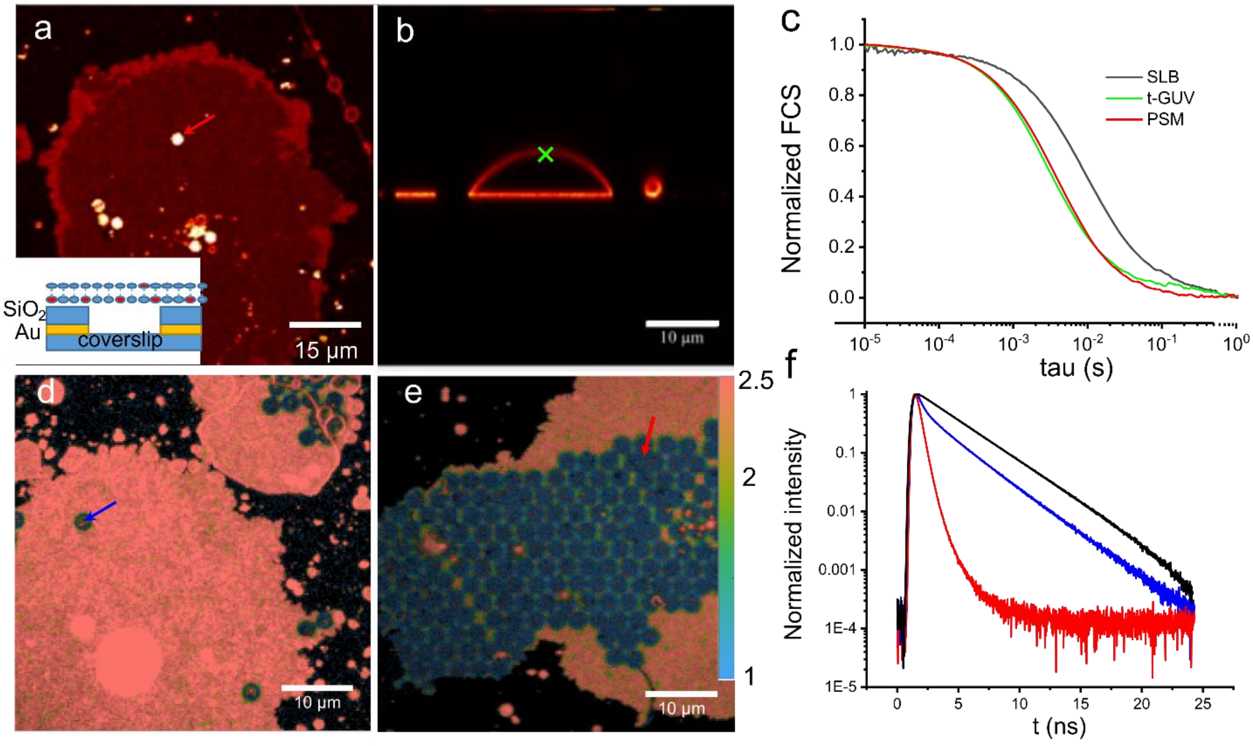

Supplementary Figure 5: Checking the generation of free-standing membranes. (a) Confocal fluorescence image showing a GUV patch on a glass-gold substrate, with an arrow indicating the position of the PSM above the glass used for FCS measurements. The inset shows a schematic representation of the structure of the substrate. (b) Confocal fluorescence image showing a vertical section of a GUV on a glass/BSA substrate, with a cross indicating the position at the top of the membrane used for FCS measurements (t-GUV). (c) FCS curves of DPPE-Atto655 for SLB, t-GUV, and PSM above glass. (d) Confocal fluorescence lifetime image showing a GUV patch on a GIET substrate, with an arrow indicating the position used for the PSM lifetime measurement. (e) Confocal fluorescence lifetime image of a GUV patch on a multi-pore graphene substrate, with an arrow indicating the position used for the lifetime measurement. (f) Fluorescence lifetime curves for a SLB on glass, for a membrane on the bottom of a pore (t-GUV), see red arrow in (e), and a PSM over a pore with 30 nm depth above a graphene substrate, see blue arrow in (d). The experiments in (a , b, d, e) were repeated three times with similar results. Source data for (c) and (f) are provided as a Source Data file.

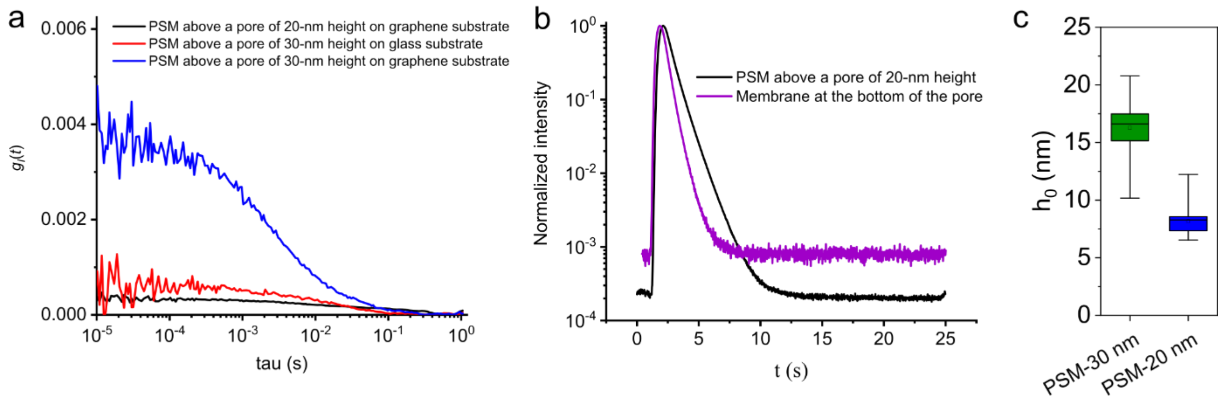

Supplementary Figure 6: Intensity correlation and lifetime decay curves for PSM measurements. (a) Intensity correlation curves $g_I(t)$ measured at the center of the PSMs above different substrates. (b) Normalized fluorescence decay curves were for a PSM above a graphene-substrate with 20 nm deep pore, and for a membrane attached to the bottom of the pore. (c) Box plot of average height values (h_0) for PSMs above prepared above pores with 30 nm and 20 nm depth. Box plots show the 25th–75th quantiles (box), median (black line), mean (black dot), and whiskers (minima to maxima). n = 20 independent measurements over 2 independent experiments for PSM-30 nm sample and n = 15 independent measurements over 2 independent experiments for PSM-20 nm sample. Source data are provided as a Source Data file.

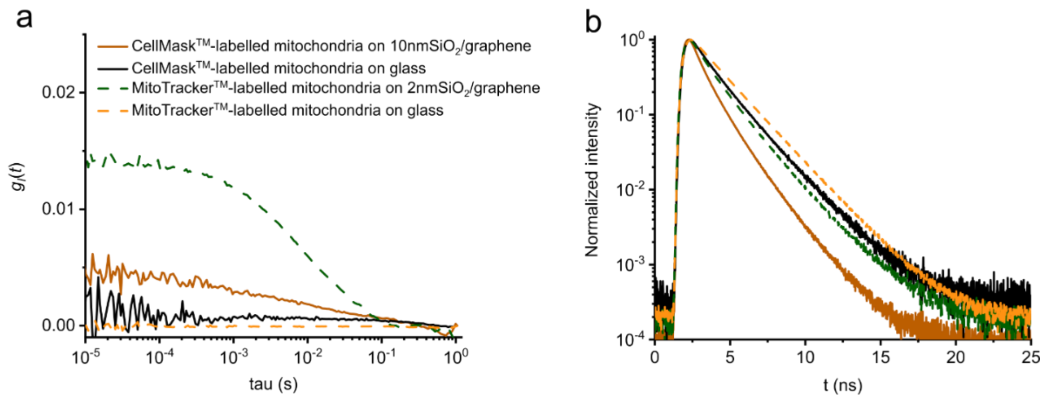

Supplementary Figure 7: Intensity correlation and lifetime decay curves for mitochondria measurements. (a) Height correlation curves, $g_h(t)$, were measured for CellMask™ and MitoTracker™ labelled mitochondria in their active state, either a MIET substrate or on a glass coverslip. (b) Corresponding fluorescence lifetime decay curves. Source data are provided as a Source Data file.

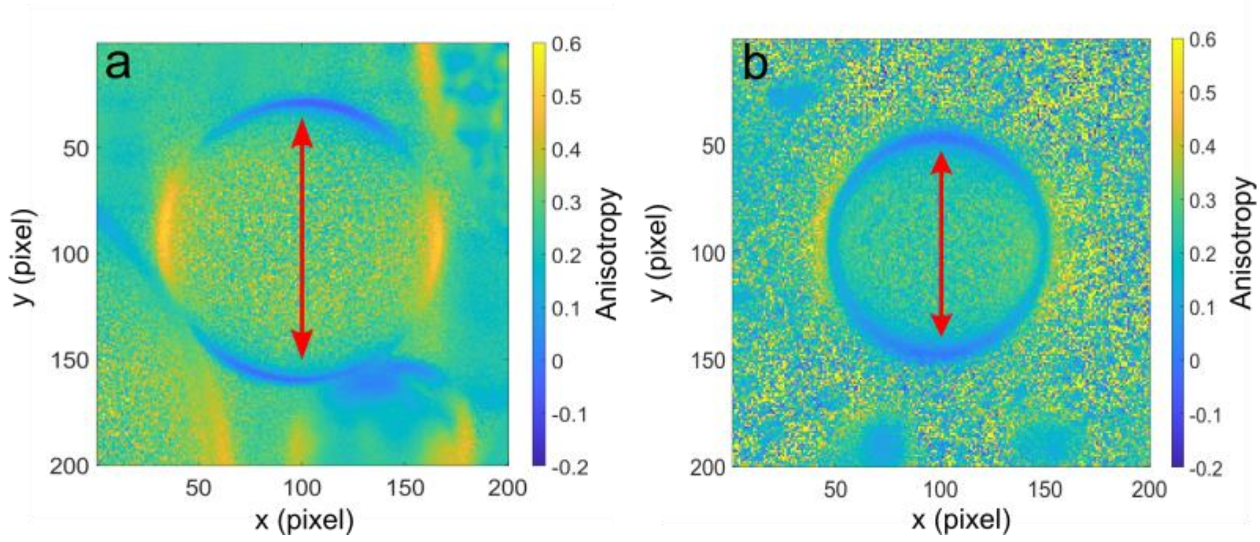

Supplementary Figure 8: Fluorescence anisotropy images of tense GUV and deflated GUV. Fluorescence anisotropy for measuring rotational mobility and orientation in (a) tense GUV and (b) deflated GUV using DPPE-atto655 lipids. The arrows show the excitation polarization. Fluorescence anisotropy was calculated as *r* = $(I_{\parallel} - I_{\perp})/(I_{\parallel} + 2I_{\perp})$, where $I_{\parallel}$ and $I_{\perp}$ are the fluorescence intensities with polarization parallel and perpendicular to the excitation polarization. The results clearly demonstrate that, in deflated GUV, the dye exhibits random orientation. This randomness arises from the fluctuations-induced deformations in lipid bilayers.[2] The experiments in (a , b) were repeated three times with similar results. For fluorescence anisotropy imaging, please refer to the detailed technical note available from PicoQuant:

(https://www.picoquant.com/?ACT=35&fid=115&d=7257&f=technote_polarization_extension_lsm.pdf).

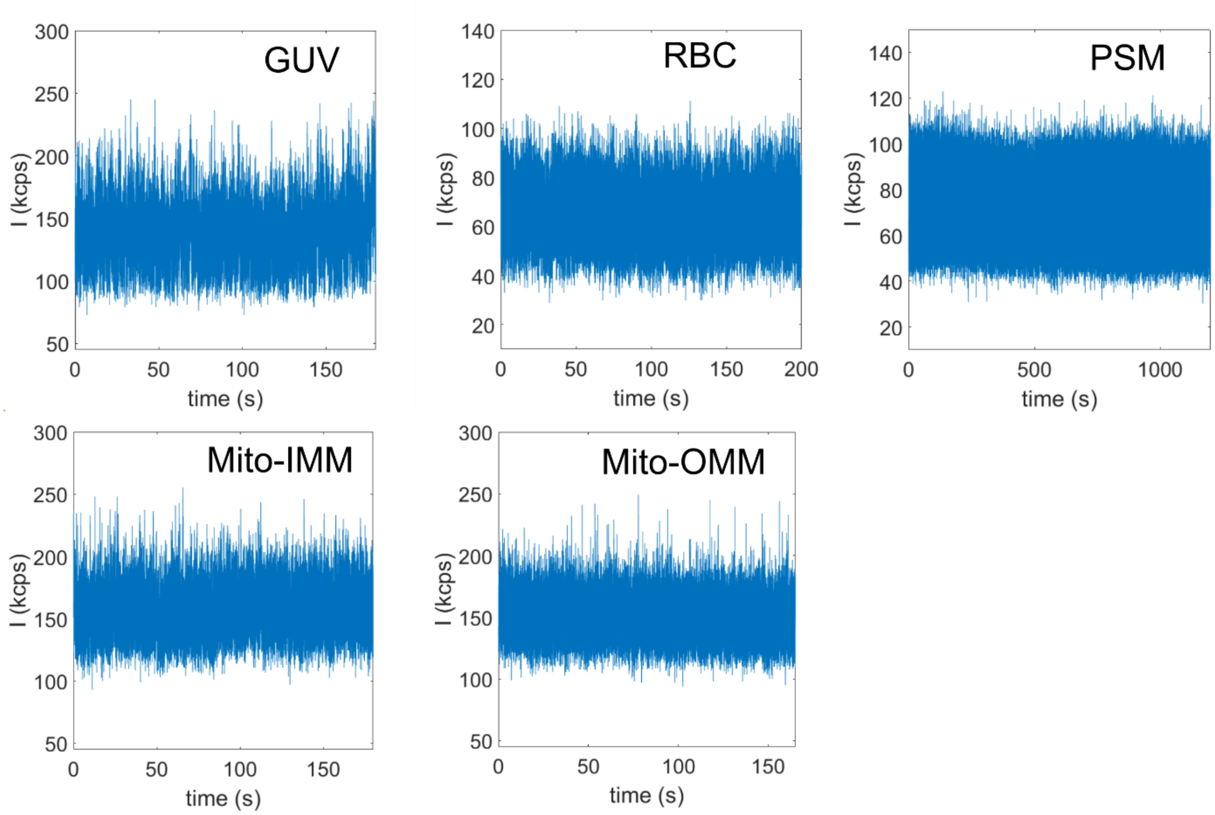

Supplementary Figure 9: Fluorescence intensity-time traces of different samples. The intensity-time traces of different samples exhibit almost no decay during the observation period, suggesting that the photobleaching effect can be safely ignored in our measurements. Source data are provided as a Source Data file.

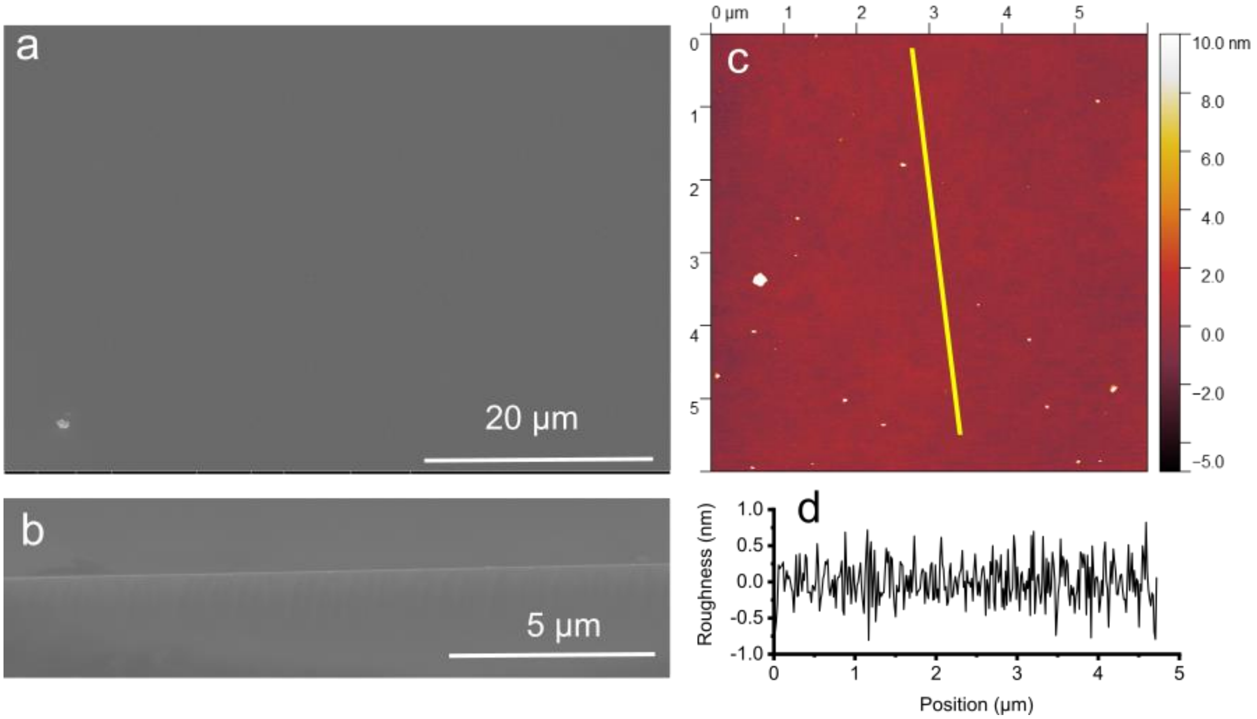

Supplementary Figure 10: AFM and SEM characterization of the MIET substrate. (a) Scanning electron microscopy (SEM) image and (b) cross-section SEM image of a MIET substrate (10 nm SiO_2/1nm Ti/10 nm Au/2 nm Ti/coverslip). (c) Atomic force image (AFM) and (d) the corresponding surface roughness profiles (red line in c) of such MIET substrate. We obtained a root-mean-square value of roughness as 1.2 nm. These surface characterizations affirm that the MIET substrate exhibits exceptional smoothness and uniformity. The experiments in (a , b) were repeated two times with similar results and the experiment in (c) was repeated three times with similar results. Source data for (d) is provided as a Source Data file.

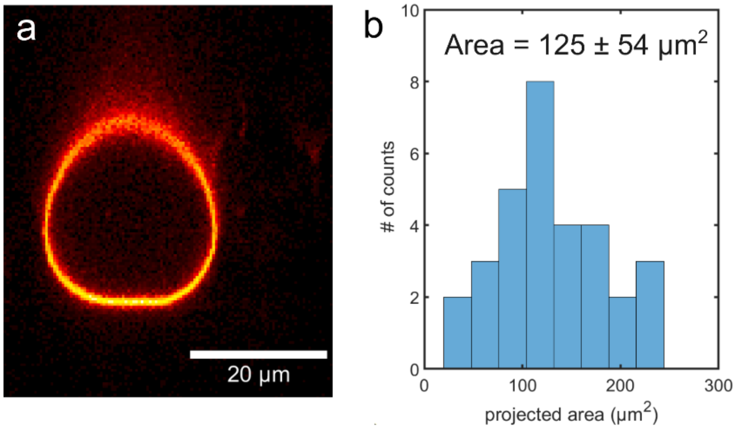

Supplementary Figure 11: xz scanning image and projected area histogram of deflated GUVs . (a) xz scanning image of one deflated GUV supported on BSA-modified glass surface, the GUV takes a non-spherical shape on the substrate. (b) The histogram of the projected areas of the deflated GUVs. The mean area is 125 ± 54 μm^2 (mean ± SD, N = 25). The experiment in (a) was repeated four times with similar results. Source data for (b) is provided as a Source Data file.

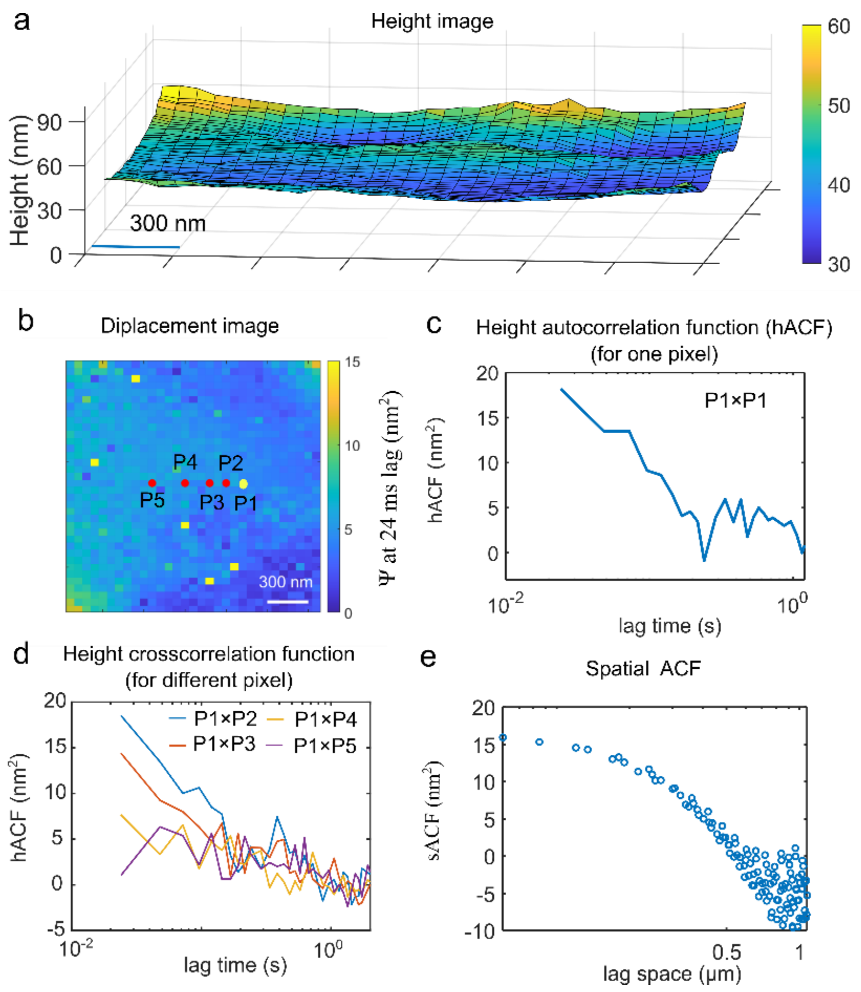

Supplementary Figure 12: Fast scanning MIET-FCS to reveal the height spatial correlation of the GUV bottom membrane. (a) A height image obtained from scanning a deflated GUV (230 mOsm/L inside and 400 mOsm/L outside) with dense labelling (1 mol%) on MIET surface. Scanning was performed at a rate of 2.4 µs per pixels for a 100 pixel × 100 pixel area (pixel size is 60 nm) and at least 9000 frames were collected. (b) The fluctuation amplitude (ψ) image at the time lag of 24 ms. The fluctuation amplitude image was obtained by building the height autocorrelation function (hACF) for each pixel (panel (c)). (c) A representative hACF for the position P1 in panel (b). (d) The height cross-correlation function (hCCF) calculated for different positions (P1 ×P2, P1 ×P3, P1 ×P4, P1 ×P5) in panel (b). (e) Representative spatial ACF was calculated from the height image for an area of 40 pixel × 40 pixel. The spatial ACF is defined as sACF $= \langle \Delta h(r')\Delta h(r'+r)\rangle$, where r is the distance lag value. Source data are provided as a Source Data file.

Supplementary Table 1: Tension and bending rigidity of RBCs obtained from different methods/techniques

Tension ($\mu J/m^2$)	Bending rigidity ($\times 10^{-20}$ J)	Method	Note	Reference
2.7 ± 2.1	3.9 ± 2.7	MIET-FCS	ATP-depleted RBC	This study
NA	2.3 ± 0.17	DPM	Healthy RBC	[3] [4] [5]
1.5 ± 0.2	0.75 ± 0.15	sHPM	Healthy RBC	[6] [7]
1.27	7.09	Light absorption imaging	Healthy RBC	[8]
0.65 ± 0.21 (ATP+)<br>1.9 ±0.1 (ATP-)	28 ± 3<br>39 ± 3	TRMFS	Healthy RBC<br>ATP-depleted RBC	[9]
0.5–1.2	NA	Theoretical calculation	NA	[10]
~ 0.5	~ 90 ± 3	Contour analysis	Healthy RBC	[11]
0.6 ± 0.19	14 ± 1.5	Optical tweezers	Healthy RBC	[12]
0.65	NA	Optical tweezers	Healthy RBC	[13]
0.5 ± 0.17	26.7 ± 4.6	Flickering analysis	Healthy RBC	[14]
NA	20.7 ± 3.2	AFM	Healthy RBC	[15]
NA	1.3 – 3	FS	Healthy RBC	[16]

DPM: diffraction phase microscopy; sHPM: stabilized Hilbert phase microscopy; TRMFS: Time resolved membrane fluctuation spectroscopy; AFM: atomic force microscopy; FS: Flicker spectroscopy.

Supplementary Note 1: Validation of MIET lifetime and brightness curves

To confirm the accuracy of the MIET lifetime and brightness curves, we conducted measurements on supported lipid bilayers (SLBs) with a well-known sample geometry. Specifically, we prepared SLBs of DOPC and 0.001% DPPE-Atto655 on four different substrates with silica spacer thickness values of 10 nm, 20 nm, 30 nm, and 50 nm. To account for the thickness of the SLBs and the hydration layer between the substrate and bottom leaflet of the SLBs, we calculated the lifetime and brightness curves assuming an average dye position of 2.5 nm above the surface (see Supplementary Figure 1a).

We took point measurements on these samples, with at least 10 measurements per sampling point. As shown in Supplementary Figure 1b and c, the measured lifetime values and count rates were found to be in excellent agreement with the theoretical calibration curves. These results confirm the accuracy of the MIET lifetime and brightness curves, and demonstrate their reliability for our experiments.

Supplementary Note 2: Pore-spanning membrane (PSM) measurements

To confirm that the pore-spanning membranes (PSMs) were free-standing, we employed two methods using negatively charged giant unilamellar vesicles (GUVs) containing DPhPC/DOPG/DPPE-Atto655 that were spanned over a positively charged pore substrate.[17] The first method involved comparing the fluorescence correlation spectroscopy (FCS) curves obtained for PSMs above a glass substrate, the top membrane of GUVs (t-GUVs), and supported lipid bilayers (SLBs), all using the same lipid mixture. The PSM above a glass is prepared on a substrate where the bottom of the pore is the bare glass substrate, while a 10 nm thick gold layer is present in the non-porous regions. Consequently, the fluorescence from the membrane on the non-porous regions is quenched by the gold layer. However, form the membrane parts suspended over the pores, fluorescence remains unquenched (Supplementary Figure 5a), which makes it easier for us to distinguish the PSMs from the GUV patch. As shown in Supplementary Figure 5c, the FCS curves for t-GUVs and PSMs were almost identical, with faster diffusion rates than that of the SLBs.

The second method involved measuring the fluorescence lifetimes of the PSMs above graphene, see Supplementary Figure 6. The lifetime of the membrane touching the bottom of the pores was found to be very short, while the free-standing membrane had a longer lifetime. It should be noted that not all PSMs were suspended above the pore bottom, and only the data with long fluorescence lifetimes were used for further analysis.

Together, these results confirm that the PSMs were free-standing and not attached to the underlying substrate.

Supplementary Note 3: Fluorescent labeling of mitochondrial membranes

To label the outer and inner mitochondrial membranes (OMM and IMM), respectively, we used commercial dyes: CellMask™ Deep Red Plasma membrane Stain (Thermo Fisher) and MitoTracker™ Deep Red FM (Thermo Fisher). We employed the highest concentrations of the dyes recommended by the manufacturer (10 µg/mL for CellMask™ and 500 nM for MitoTracker™) to ensure optimal membrane labeling.

To confirm that these concentrations were sufficient to suppress fluorescence fluctuations due to lateral diffusion, we first measured the correlation function of the labeled mitochondria on glass substrates. As shown in Supplementary Figure 7a, the small amplitudes found on a glass substrate as compared to the larger amplitudes observed on a graphene substrate indicate that the lateral diffusion-related fluctuations originating from labeled OMM and IMM can be ignored. This confirms that the chosen dye concentrations are appropriate for our experiments and enables accurate interpretation of the results.

Supplementary Note 4: Fitting the height correlation function of ATP-depleted RBCs with eq 4

We employed the equilibrium theories described by eq 4 to fit the height correlation function of ATP-depleted RBCs. In contrast to GUV, we did not maintain the bending rigidity, *k*, as a constant during the fitting of the height correlation function due to the broad distribution of published values for the k of RBCs (see Supplementary Table 1). The effective viscosity, η, is calculated as the arithmetic mean of the viscosity of the RBCs (5.5 mPas)[3] and the outer buffer (1 mPas).[18,19] The fitting determined the tension σ (= 2.7 ± 2.1 µJ/m^2 N = 22), the interaction potential strength γ (= 6.6 ± 6.1 MJ·m^{-4}), and the bending rigidity *k* (= (3.9 ± 2.7) × 10^{-20} J).

Supplementary References

(1) Schneider, F.; Ruhlandt, D.; Gregor, I.; Enderlein, J.; Chizhik, A. I. Quantum Yield Measurements of Fluorophores in Lipid Bilayers Using a Plasmonic Nanocavity. *J. Phys. Chem. Lett.* **2017**, *8* (7), 1472–1475. https://doi.org/10.1021/acs.jpclett.7b00422.

(2) Brannigan, G.; Brown, F. L. H. A Consistent Model for Thermal Fluctuations and Protein-Induced Deformations in Lipid Bilayers. *Biophysical Journal* **2006**, *90* (5), 1501–1520. https://doi.org/10.1529/biophysj.105.075838.

(3) Byun, H.; Hillman, T. R.; Higgins, J. M.; Diez-Silva, M.; Peng, Z.; Dao, M.; Dasari, R. R.; Suresh, S.; Park, Y. Optical Measurement of Biomechanical Properties of Individual Erythrocytes from a Sickle Cell Patient. *Acta Biomaterialia* **2012**, *8* (11), 4130–4138. https://doi.org/10.1016/j.actbio.2012.07.011.

(4) Park, Y.; Best, C. A.; Badizadegan, K.; Dasari, R. R.; Feld, M. S.; Kuriabova, T.; Henle, M. L.; Levine, A. J.; Popescu, G. Measurement of Red Blood Cell Mechanics during Morphological Changes. *Proceedings of the National Academy of Sciences* **2010**, *107* (15), 6731–6736. https://doi.org/10.1073/pnas.0909533107.

(5) Park, Y.; Best, C. A.; Kuriabova, T.; Henle, M. L.; Feld, M. S.; Levine, A. J.; Popescu, G. Measurement of the Nonlinear Elasticity of Red Blood Cell Membranes. *Phys. Rev. E* **2011**, *83* (5), 051925. https://doi.org/10.1103/PhysRevE.83.051925.

(6) Popescu, G.; Park, Y.; Choi, W.; Dasari, R. R.; Feld, M. S.; Badizadegan, K. IMAGING RED BLOOD CELL DYNAMICS BY QUANTITATIVE PHASE MICROSCOPY. *Blood Cells Mol Dis* **2008**, *41* (1), 10–16. https://doi.org/10.1016/j.bcmd.2008.01.010.

(7) Popescu, G.; Ikeda, T.; Goda, K.; Best-Popescu, C. A.; Laposata, M.; Manley, S.; Dasari, R. R.; Badizadegan, K.; Feld, M. S. Optical Measurement of Cell Membrane Tension. *Phys. Rev. Lett.* **2006**, *97* (21), 218101. https://doi.org/10.1103/PhysRevLett.97.218101.

(8) Paul, R.; Zhou, Y.; Nikfar, M.; Razizadeh, M.; Liu, Y. Quantitative Absorption Imaging of Red Blood Cells to Determine Physical and Mechanical Properties. *RSC Advances* **2020**, *10* (64), 38923–38936. https://doi.org/10.1039/D0RA05421F.

(9) Betz, T.; Lenz, M.; Joanny, J.-F.; Sykes, C. ATP-Dependent Mechanics of Red Blood Cells. *PNAS* **2009**, *106* (36), 15320–15325. https://doi.org/10.1073/pnas.0904614106.

(10) Gov, N. S.; Safran, S. A. Red Blood Cell Membrane Fluctuations and Shape Controlled by ATP-Induced Cytoskeletal Defects. *Biophys J* **2005**, *88* (3), 1859–1874. https://doi.org/10.1529/biophysj.104.045328.

(11) Evans, J.; Gratzer, W.; Mohandas, N.; Parker, K.; Sleep, J. Fluctuations of the Red Blood Cell Membrane: Relation to Mechanical Properties and Lack of ATP Dependence. *Biophysical Journal* **2008**, *94* (10), 4134–4144. https://doi.org/10.1529/biophysj.107.117952.

(12) Kariuki, S. N.; Marin-Menendez, A.; Introini, V.; Ravenhill, B. J.; Lin, Y.-C.; Macharia, A.; Makale, J.; Tendwa, M.; Nyamu, W.; Kotar, J.; Carrasquilla, M.; Rowe, J. A.; Rockett, K.; Kwiatkowski, D.; Weekes, M. P.; Cicuta, P.; Williams, T. N.; Rayner, J. C. Red Blood Cell Tension Protects against Severe Malaria in the Dantu Blood Group. *Nature* **2020**, *585* (7826), 579–583. https://doi.org/10.1038/s41586-020-2726-6.

(13) Turlier, H.; Fedosov, D. A.; Audoly, B.; Auth, T.; Gov, N. S.; Sykes, C.; Joanny, J.-F.; Gompper, G.; Betz, T. Equilibrium Physics Breakdown Reveals the Active Nature of Red Blood Cell Flickering. *Nature Phys* **2016**, *12* (5), 513–519. https://doi.org/10.1038/nphys3621.

(14) Yoon, Y.-Z.; Hong, H.; Brown, A.; Kim, D. C.; Kang, D. J.; Lew, V. L.; Cicuta, P. Flickering Analysis of Erythrocyte Mechanical Properties: Dependence on Oxygenation Level, Cell Shape, and Hydration Level. *Biophys J* **2009**, *97* (6), 1606–1615. https://doi.org/10.1016/j.bpj.2009.06.028.

(15) Scheffer, L.; Bitler, A.; Ben-Jacob, E.; Korenstein, R. Atomic Force Pulling: Probing the Local Elasticity of the Cell Membrane. *Eur Biophys J* **2001**, *30* (2), 83–90. https://doi.org/10.1007/s002490000122.
(16) Brochard, F.; Lennon, J. F. Frequency Spectrum of the Flicker Phenomenon in Erythrocytes. *J. Phys. France* **1975**, *36* (11), 1035–1047. https://doi.org/10.1051/jphys:01975003601101103500.
(17) Heinemann, F.; Schwille, P. Preparation of Micrometer-Sized Free-Standing Membranes. *ChemPhysChem* **2011**, *12* (14), 2568–2571. https://doi.org/10.1002/cphc.201100438.
(18) Monzel, C.; Schmidt, D.; Kleusch, C.; Kirchenbüchler, D.; Seifert, U.; Smith, A.-S.; Sengupta, K.; Merkel, R. Measuring Fast Stochastic Displacements of Bio-Membranes with Dynamic Optical Displacement Spectroscopy. *Nat Commun* **2015**, *6* (1), 8162. https://doi.org/10.1038/ncomms9162.
(19) Monzel, C.; Schmidt, D.; Seifert, U.; Smith, A.-S.; Merkel, R.; Sengupta, K. Nanometric Thermal Fluctuations of Weakly Confined Biomembranes Measured with Microsecond Time-Resolution. *Soft Matter* **2016**, *12* (21), 4755–4768. https://doi.org/10.1039/C6SM00412A.
