# Supplementary material for: Measuring sub-nanometer undulations at microsecond temporal resolution with metal- and graphene-induced energy transfer spectroscopy
Source: Nat Commun. 2024 Feb 27;15:1789. doi: 10.1038/s41467-024-45822-x (PMC10899616; doi:10.1038/s41467-024-45822-x)
Supplement: Supplementary file 3 — Reporting Summary [file 41467_2024_45822_MOESM3_ESM.pdf]

nature portfolio

Corresponding author(s): Jörg Enderlein

Last updated by author(s): Jan 12, 2024

Reporting Summary

Nature Portfolio wishes to improve the reproducibility of the work that we publish. This form provides structure for consistency and transparency in reporting. For further information on Nature Portfolio policies, see our Editorial Policies and the Editorial Policy Checklist.

Statistics

For all statistical analyses, confirm that the following items are present in the figure legend, table legend, main text, or Methods section.

n/a	Confirmed	
☐	☒	The exact sample size (*n*) for each experimental group/condition, given as a discrete number and unit of measurement
☐	☒	A statement on whether measurements were taken from distinct samples or whether the same sample was measured repeatedly
☒	☐	The statistical test(s) used AND whether they are one- or two-sided<br>*Only common tests should be described solely by name; describe more complex techniques in the Methods section.*
☒	☐	A description of all covariates tested
☒	☐	A description of any assumptions or corrections, such as tests of normality and adjustment for multiple comparisons
☐	☒	A full description of the statistical parameters including central tendency (e.g. means) or other basic estimates (e.g. regression coefficient) AND variation (e.g. standard deviation) or associated estimates of uncertainty (e.g. confidence intervals)
☒	☐	For null hypothesis testing, the test statistic (e.g. *F*, *t*, *r*) with confidence intervals, effect sizes, degrees of freedom and *P* value noted<br>*Give P values as exact values whenever suitable.*
☒	☐	For Bayesian analysis, information on the choice of priors and Markov chain Monte Carlo settings
☒	☐	For hierarchical and complex designs, identification of the appropriate level for tests and full reporting of outcomes
☒	☐	Estimates of effect sizes (e.g. Cohen's *d*, Pearson's *r*), indicating how they were calculated

Our web collection on statistics for biologists contains articles on many of the points above.

Software and code

Policy information about availability of computer code

Data collection	SymphoTime softaware (v. 2.8) by PicoQuant GmbH, Berlin (software accompanying their MicroTime single-molecule spectroscopy systems: https://www.picoquant.com/products/category/software/symphotime-64-fluorescence-lifetime-imaging-and-correlation-software)
Data analysis	Matlab v. 2022b (analyzing code: https://gitlab.gwdg.de/igregor/miet-membrane-fluctuation.git; a Readme file in the repository to explain all the analysis details);<br>Mathematica v. 13.2.1.0 (https://gitlab.gwdg.de/igregor/miet-membrane-fluctuation.git);<br>TrackNTrace Lifetime Edition (https://github.com/scstein/TrackNTrace)

For manuscripts utilizing custom algorithms or software that are central to the research but not yet described in published literature, software must be made available to editors and reviewers. We strongly encourage code deposition in a community repository (e.g. GitHub). See the Nature Portfolio guidelines for submitting code & software for further information.

Data

Policy information about availability of data

All manuscripts must include a data availability statement. This statement should provide the following information, where applicable:

- Accession codes, unique identifiers, or web links for publicly available datasets
- A description of any restrictions on data availability
- For clinical datasets or third party data, please ensure that the statement adheres to our policy

Source data are provided with this paper. There are no restrictions on data availability. All data supporting the findings of this study are available within the main text, supplementary information, and 'Source data' Excel file. Two unprocessed raw data files are also deposited in the GitHub database at https://gitlab.gwdg.de/igregor/miet-membrane-fluctuation.git. Upon reasonable request, all raw data files can be requested from the first author Dr. Tao Chen (tao.chen@phys.uni-goettingen.de).

Research involving human participants, their data, or biological material

Policy information about studies with human participants or human data. See also policy information about sex, gender (identity/presentation), and sexual orientation and race, ethnicity and racism.

Reporting on sex and gender	The sex and gender were not considered in study design because the primary focus of the study doesn't involve investigating differences related to sex or gender, conducting separate analyses might not align with the research objectives.
Reporting on race, ethnicity, or other socially relevant groupings	N/A
Population characteristics	The red blood cell donor is a healthy male individual aged 34.
Recruitment	The red blood cell donor was a volunteer. The donor provided informed consent to have the samples collected and used for research.
Ethics oversight	Biosampling was conducted according to the Declaration of Helsinki and according to the general instructions of the ethics committee of the University of Göttingen Faculty of Medicine, Göttingen, Germany. No ethics approval was judged necessary, since the procedure did not involve ethically-relevant issues.

Note that full information on the approval of the study protocol must also be provided in the manuscript.

Field-specific reporting

Please select the one below that is the best fit for your research. If you are not sure, read the appropriate sections before making your selection.

☒ Life sciences ☐ Behavioural & social sciences ☐ Ecological, evolutionary & environmental sciences

For a reference copy of the document with all sections, see nature.com/documents/nr-reporting-summary-flat.pdf

Life sciences study design

All studies must disclose on these points even when the disclosure is negative.

Sample size	No statistical method was used to predetermine sample size, and the sample size was based on the similar studies published before (Monzel, et al. Nat. Commun. 2015, 6:8162).
Data exclusions	No data were excluded from the analyses.
Replication	All experiments were repeated at least twice with reproducible results.
Randomization	In this study, randomization wasn't applicable in the traditional sense as there was no random assignment of participants to different groups or treatments. Instead, the focus was on analyzing measurements or samples taken from a single source. For the measured red blood cells samples, these measurements were undergoes randomization in their collecting and analysis to minimize biases, the study didn't involve assigning individuals to different experimental conditions.
Blinding	In this study, blinding wasn't applicable because it involved analyzing red blood cells from a single healthy individual (first author Dr. Tao Chen), without any treatments or interventions administered.

Reporting for specific materials, systems and methods

We require information from authors about some types of materials, experimental systems and methods used in many studies. Here, indicate whether each material, system or method listed is relevant to your study. If you are not sure if a list item applies to your research, read the appropriate section before selecting a response.

Materials & experimental systems

n/a	Involved in the study
☒	☐ Antibodies
☐	☒ Eukaryotic cell lines
☒	☐ Palaeontology and archaeology
☒	☐ Animals and other organisms
☒	☐ Clinical data
☒	☐ Dual use research of concern
☒	☐ Plants

Methods

n/a	Involved in the study
☒	☐ ChIP-seq
☒	☐ Flow cytometry
☒	☐ MRI-based neuroimaging

Eukaryotic cell lines

Policy information about cell lines and Sex and Gender in Research

Cell line source(s)	MHS cell (ATCC)
Authentication	All cell lines were authenticated by providers.
Mycoplasma contamination	We performe regular testing for mycoplasma contamination. All cell lines were negative.
Commonly misidentified lines (See ICLAC register)	No commonly misidentified lines were used in this study.

Plants

Seed stocks	N/A
Novel plant genotypes	N/A
Authentication	N/A
